# A misdiagnosed infection mimicking “tree man disease”

**DOI:** 10.1371/journal.pntd.0005543

**Published:** 2017-06-15

**Authors:** Jo Anne Lim, Zulrusydi Ismail, Che Noraini Ibrahim, Soon Eu Chong, Wan Noor Hasbee Wan Abdullah

**Affiliations:** 1 Department of Dermatology, Hospital Raja Perempuan Zainab II, Kota Bharu, Kelantan, Malaysia; 2 Cluster of Regenerative Medicine, Advanced Medical and Dental Institute, Universiti Sains Malaysia, Penang, Malaysia; University of California San Diego School of Medicine, UNITED STATES

## Case description

**Fig 1:**
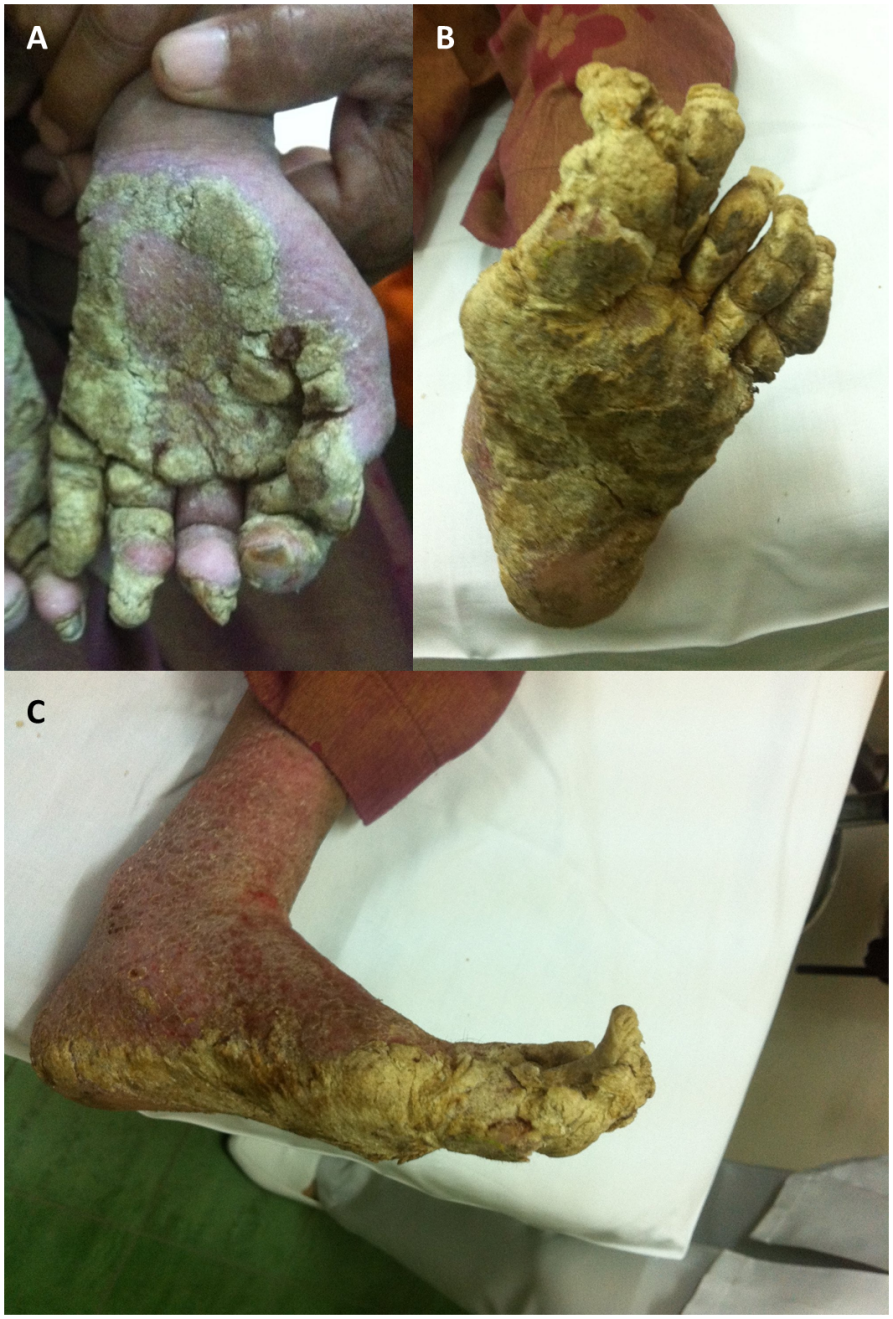
Extensive generalized hyperkeratotic plaques over the hands and feet. **(A)** Left hand palmar view. **(B)** Left foot plantar view. **(C)** Left foot medial view.

**Fig 2:**
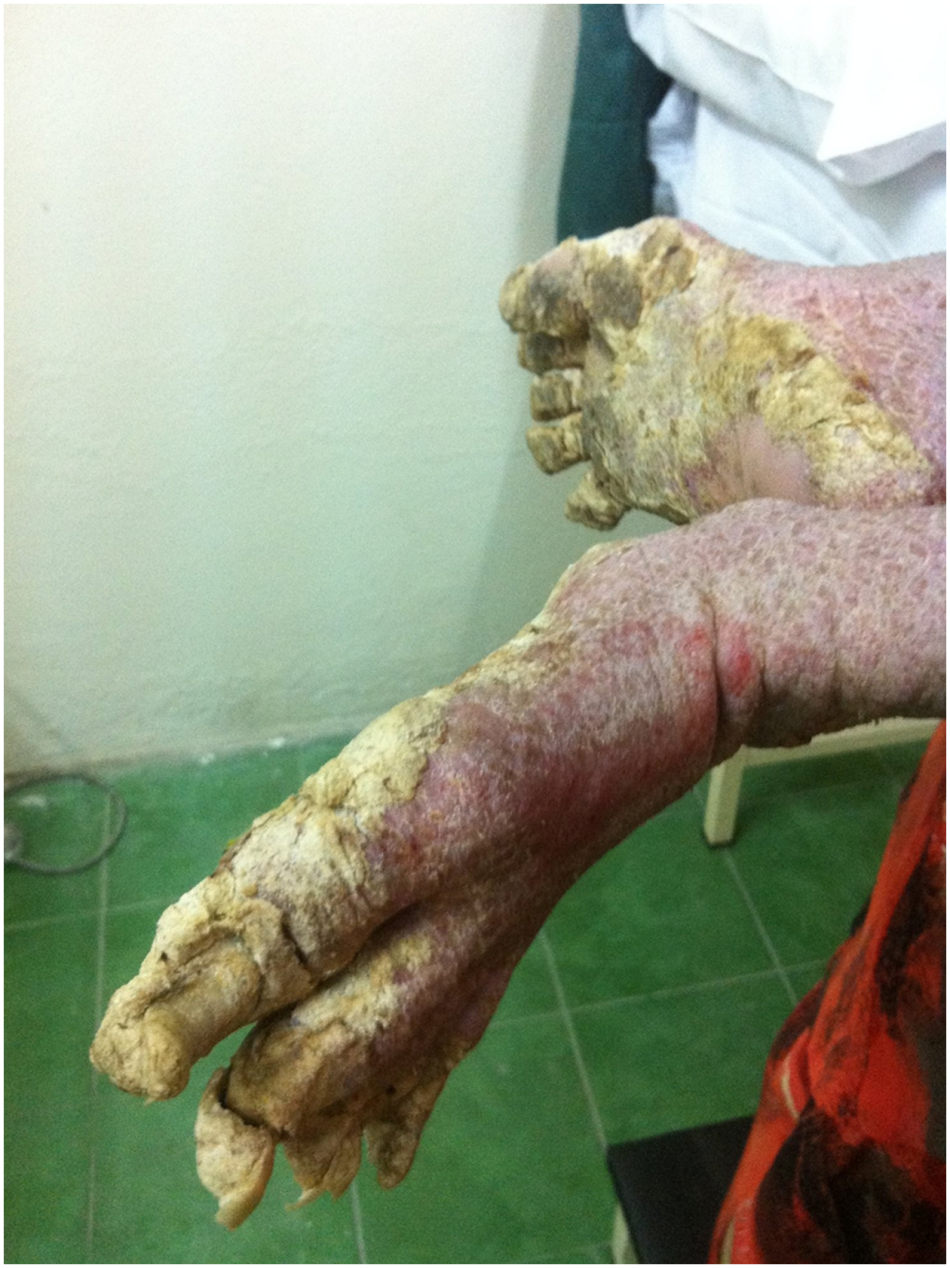
The deformed crusted plaques over the lower extremities made her unable to walk.

A 10-year-old girl with Down syndrome in Malaysia presented with extensive and generalized hyperkeratotic papules and plaques on her entire body (Fig 1) over a 4-year period. There was mild itching with severe hand and foot deformity. She was socially withdrawn with poor self-esteem and poor hygiene. She was also malnourished and could neither feed herself nor walk due to the deformity of her extremities (Fig 2).

The girl’s entire family also had prolonged itchy skin papules, but they were less severe, without any hyperkeratotic plaques or deformity. With a poor socioeconomic background, they had lived in an overcrowded house.

The patient lived in a village with the nearest hospital a 3-hour drive away and was taken to multiple small district clinics and traditional healers. She was misdiagnosed with eczema, psoriasis, and fungal infection. Due to the remote location of their home and failed treatments due to incorrect diagnosis, the parents had given up hope seeking treatment and assumed their child had a condition known locally as “orang pokok” or “tree man” (epidermodysplasia verruciformis [EV]), a condition that received widespread media attention several years ago.

Resigned to fate and needing financial aid, the family of the patient opted for public donation via the local newspaper. When her case was published, it attracted the attention of the state department of health, and a home visit was done by a dermatology team to investigate.

## Diagnosis of Norwegian scabies, clinical course, and treatment

**Fig 3:**
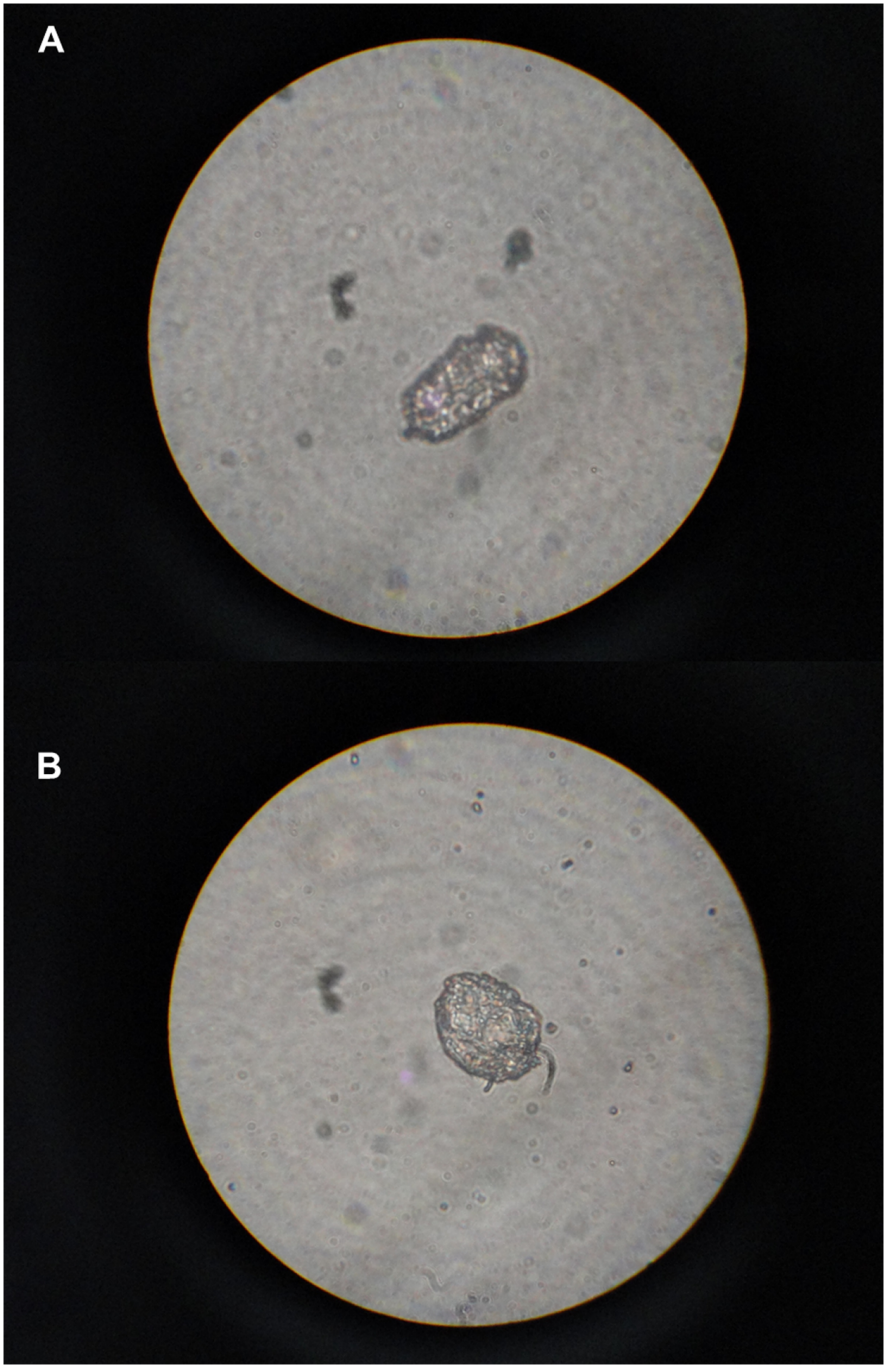
Skin scraping on microscopy. **(A)** scabies ova, **(B)** scabies mites.

Skin scraping in potassium hydroxide mount revealed *Sarcoptes scabiei* adult mites, eggs, and faecal pellets (Fig 3). Hence she was diagnosed to have Norwegian scabies.

The patient was treated with weekly permethrin plus daily therapy of benzyl benzoate emulsion alternating with salicylic acid ointment, both under occlusion. This eventually resulted in a complete resolution within 2 weeks of treatment. Oral Augmentin and topical fucidic acid cream were also given to treat areas with superimposed bacterial infection. All contacts were treated, and she was referred to social welfare department for financial support. Posttreatment, she can finally feed herself and is able to attend special schooling.

## Discussion

Norwegian scabies is a very contagious variant of scabies in which there are millions of mites but surprisingly little itch [1]. Unlike the usual form of scabies, it may affect the scalp and may mimic EV. Scabies becomes severely crusted in individuals with poor immune systems, the malnourished, the elderly, and those with intellectual disability, cognitive deficiency, or physical disabilities, who are unable to scratch or interpret the itch [2]. According to the severity-grading scale for crusted scabies by Davis et al. [3], this patient fulfilled the criteria for grade 3 crusted scabies. The crusting was so severe that the family thought it was a condition called “tree man” (or EV), which received media attention.

EV is a rare autosomal recessive skin disorder characterized by abnormal susceptibility to human papillomavirus (HPV) infection, causing eruptions of wart-like lesions that are at risk of malignant transformation [4]. Unlike Norwegian scabies, the papules and skin lesions are non-itchy. To date, there has been no effective curative treatment against EV [5]. This disease was made known in the mainstream media among the local population when an Indonesian man was reported to have excessive growth of warts forming tree-like lesions on the hands and feet [6].

Norwegian scabies is associated with an increased morbidity and mortality [7, 8]. Scratching may predispose a patient to secondary bacterial infection and sepsis [9]. The diagnosis of scabies is often missed initially by doctors because of its variable presentations [9, 10]. Nevertheless, the symptom of itching should have raised the suspicion of such a condition by a general practitioner. This particular patient is intellectually challenged and thus unable to express or respond to her symptoms. The tell-tale sign of her condition is the presence of itching among family members. A high index of suspicion and good observation is required to detect this treatable disease. Any further delay in treating this condition could have led to severe infection, sepsis, and even death. Wrong topical application technique, untreated contacts and improper fomite decontamination can also lead to treatment failure at the primary care level.

Oral ivermectin is another treatment option for such a patient with extensive crusted lesions which require prolonged treatment involving tedious topical antiscabetic applications. It was not given in this case, as this medication is currently not available in Malaysia. Oral ivermectin can also benefit patients who are bedbound or who have restricted mobility, as topical treatment cannot be applied properly in these cases.

## Conclusion

This presentation of Norwegian scabies emphasizes the need to include scabies as a differential diagnosis when a patient is presented with crusted cutaneous lesions. Early diagnosis and proper treatment is crucial to avoid unwanted morbidity and mortality.

## Ethics statements

The patient’s parent has given written consent for this publication. Institutional review board approval is not required at our institution for the reporting of a single case.

**Key learning points**

- The diagnosis of scabies can be missed initially by doctors because of its variable presentations.
- Scabies may be an underdiagnosed infectious disease among the intellectually challenged.
- General practitioners need to include scabies as a differential diagnosis when a patient is presented with crusted cutaneous lesion.

## Supporting information

S1 Checklist STARD checklist. (DOCX)
